# Toward a Circular Bioeconomy: Exploring Pineapple Stem Starch Film as Protective Coating for Fruits and Vegetables

**DOI:** 10.3390/polym15112493

**Published:** 2023-05-29

**Authors:** Krongkarn Bumrungnok, Poonsub Threepopnatkul, Taweechai Amornsakchai, Chin Hua Chia, Rungtiwa Wongsagonsup, Siwaporn Meejoo Smith

**Affiliations:** 1 Center of Sustainable Energy and Green Materials, Faculty of Science, Mahidol University, Phuttamonthon 4 Road, Salaya, Nakhon Pathom 73170, Thailandsiwaporn.smi@mahidol.ac.th (S.M.S.); 2 Department of Materials Science and Engineering, Faculty of Engineering and Industrial Technology, Silpakorn University, Nakhon Pathom 73000, Thailand; 3 Department of Applied Physics, Faculty of Science and Technology, Universiti Kebangsaan Malaysia, Bangi 43600, Selangor, Malaysia; chia@ukm.edu.my; 4 Division of Food Technology, Kanchanaburi Campus, Mahidol University, Kanchanaburi 71150, Thailand; rungtiwa.won@mahidol.ac.th; 5 Food and Nutrition Academic and Research Cluster, Institute of Nutrition, Mahidol University, Nakhon Pathom 73170, Thailand

**Keywords:** pineapple stem, agricultural waste, starch, amylose, coating, circular economy

## Abstract

In order to reduce our dependence on nonrenewable plastics and solve the problem of non-biodegradable plastic waste, there has been much attention paid to the development of biodegradable plastics from natural resources. Starch-based materials have been widely studied and developed for commercial production, primarily from corn and tapioca. However, the use of these starches could generate food security problems. Therefore, the use of alternative starch sources, such as agricultural waste, would be of great interest. In this work, we investigated the properties of films prepared from pineapple stem starch, which has a high amylose content. Pineapple stem starch (PSS) films and glycerol-plasticized PSS films were prepared and characterized using X-ray diffraction and water contact angle measurements. All films exhibited some degree of crystallinity, making them water-resistant. The effect of glycerol content on mechanical properties and gas (oxygen, carbon dioxide and water vapor) transmission rates was also studied. The tensile modulus and tensile strength of the films decreased with increasing glycerol content, while gas transmission rates increased. Preliminary studies showed that coatings made from PSS films could slow down the ripening process of bananas and extend their shelf life.

## 1. Introduction

One of the top greenhouse gas (GHG) emitters is the food supply chain [1]. Whether or not the food produced is consumed, GHGs are already generated at every step of the food supply system. It was estimated that about 14% of the world’s food is lost between harvest and retail and about 17% is wasted in retail and at the consumption level and these account for 8–10% of global GHGs [1]. Thus, there is a need to reduce this loss and waste. One way is to extend the shelf life of produce (fruits and vegetables) by keeping it fresh by slowing down the ripening process and preventing spoilage from microbial activity [2]. These effects could be achieved by using active packaging [3,4,5,6]. Active packaging could range from using conventional plastic bags with a sachet of active absorbers [3] to film incorporated with an active agent [7,8,9]. The use of plant-based materials either as packaging film or active agent has become a topic of interest [6,10,11,12]. However, many of these materials would not keep up with the circular and sustainable trends. Therefore, a coating that is made from edible materials has been studied as an alternative to replace packaging. In addition, the coating would provide a good appearance with a shiny skin and improve water resistance of the product, which would help extend its shelf life. There have been a number of studies on starch-based coating, for example [6,13,14,15,16,17]. The drawback of starch-based coating agents, however, is their lack of mechanical strength, hydrophilicity and brittleness [18]. Several solutions have been suggested to address these issues, including blending with cationic starch [14], chemical modification [15,16] and blending with film forming polymers, see for example [19,20,21,22,23,24,25]. Chemical modifications of starch or blending with film-forming polymers not only complicate the process but also result in products with greater material and energy intensities as well as a larger carbon footprint. Therefore, finding a starch that requires little or no modification and could form a good film would be a more sustainable route. If the starch could be derived from waste biomass, this would lead to even more sustainability.

Different non-conventional or non-food starches have been researched [26,27,28,29,30]. However, to the best of our knowledge, only starch from mango kernel has been studied for coating to extend the shelf life of agricultural products [31,32,33]. Pineapple stem is another non-conventional source of starch that is abundantly available and has not been fully utilized [34,35].

Therefore, the objective of this work is to develop an edible and biodegradable coating material from pineapple stem starch (PSS). Unlike other starch sources, PSS has been found to possess a higher amylose content [36] and it can form films of relatively high strength without having to be blended with another film-forming polymer [36]. Despite this advantage, PSS has not been studied as a fruit coating agent. This work aims to investigate the water resistance, strength and gas transmission rate of PSS films, as well as the effects of plasticizer on their properties. We also demonstrate the potential of PSS films as a coating material to prolong the shelf life of bananas.

## 2. Materials and Methods

### 2.1. Materials

Pineapple stem waste, a byproduct from a proprietary bromelain extraction process, was obtained from a local source (Hong Mao Biochem, Rayong, Thailand). In brief, the process involved crushing peeled pineapple stems to disrupt the cell structure and the liquid was extracted by centrifugation [37]. The remaining solid material was dried under the sun for a few days and further ground into powder using a food grinder. The stem powder was collected by sieving (80 mesh) to separate coarse fibers, cell walls and other solid contaminants which constitute about 56% of the whole mass. The powder contained about 15 wt.% of extractive constituents. The characteristics of the powder were similar to those obtained by wet milling, reported previously [35]. Analytical reagent grade (99.5%) glycerol was obtained from KemAus in Australia.

### 2.2. Methods

#### 2.2.1. Extraction of Pineapple Stem Starch

Pineapple stem powder was soaked in reverse osmosis (RO) water. Then, the suspension was filtered to remove unwanted fibers through a muslin cloth. After that, the suspension was filtered through a 400-mesh sieve to eliminate contaminating particles. The suspension was left for 1 h to allow the starch particles to settle at the bottom of the container. The supernatant was discarded. The starch paste was washed with RO water at least 3 times to eliminate any water-soluble substances. The paste was dried in a hot air oven at 60°C for 24 h. The PSS was crushed into fine particles of size 30–40 μm.

#### 2.2.2. Preparation of Starch Films

Starch films were produced using a solution casting method, while glycerol was used as plasticizer to produce plasticized PSS films. Briefly, the PSS powder was gelatinized by heating a PSS suspension in RO water with ratio 1:5 (g PSS: mL RO water) on a magnetic stirrer hot plate until it reached 90 °C. The heating and stirring processes were continued for 30 min. After that, the gelatinized mixture was cooled down to 40 °C and casted in petri dishes and dried in the hot air oven at 40 °C for 12 h to produce films with a thickness of around 70–90 μm. The films were peeled off from the petri dishes and kept in a desiccator for further experiments. To produce plasticized PSS films, glycerol was dissolved in RO water before the gelatinization. Plasticized PSS films were produced by adding glycerol at 10, 15, 20 and 25 wt.% of total mass (excluding water). The same experimental procedures were followed to produce plasticized PSS films. Table 1 displays the composition of the films studied and their corresponding codes.

#### 2.2.3. Characterizations

Film Transparency: The transparency of the PSS and plasticized PSS films were observed at 550 nm with a UV-VIS spectrophotometer (UV-1800, Shimadzu, Japan). Moreover, the appearance of the films was captured on photographs.

X-ray diffraction (XRD): X-ray diffraction patterns of the films were obtained from a benchtop X-ray powder diffractometer (D2 Phaser, Bruker, Germany) using an X-ray wavelength of 1.54 Å with a step scan of 15 s/point over the 2θ of 5–40 degrees. The percentage crystallinity of each PSS film sample was determined using Equation (1) [38].

(1) $$\mathrm{Crystallinity}\;(\%)=\frac{A_{c}}{A_{c}+A_{a}}\times 100$$

where

1. A_c_ = the area of crystalline region.
2. A_a_ = the area of amorphous region.

Mechanical properties: Tensile properties of the PSS and plasticized PSS films were measured on a universal testing machine (Instron 5969, Instron Engineering Corporation, Norwood, MA, USA) following ASTM D882. Rectangular specimens with size 10 × 70 mm^2^ were cut from the films and tested with an extension rate of 5 mm/min using a 5 kN load cell.

Morphology: Surfaces and fractured surfaces of PSS and plasticized PSS films were observed with a scanning electron microscope (SEM) (JSM-IT500, JEOL, Chiyoda, Japan). The samples were coated with platinum before the observation.

Gas transmission rate (GTR): Oxygen and carbon dioxide transmission rates of the films were determined using differential pressure methods (manometric methods) according to ISO 15105-1 with a gas transmission tester (GDP-C, Brugger Feinmechanik GmbH, Germany). Film specimens were placed in the instrument with aluminum foil to limit the gas transmission area to 5.064 cm^2^. The measurements were performed with a gas flow rate of 50 cm^3^/min and 0% relative humidity for 1.5 h. After that, oxygen and carbon dioxide transmission rates were normalized with the film thickness using Equation (2).

OTR or CO_2_TR = GTR × l (2)

where OTR and CO_2_TR are oxygen transmission rate and carbon dioxide transmission rate (mm·cm^3^/m^2^·d·bar), respectively. GTR is the gas transmission rate (cm^3^/m^2^·d·bar) from the gas transmission tester and l is the film thickness (mm).

Water Vapor Transmission rate (WVTR): the Dry Cup or desiccant method as described in ASTM E96 was used to determine the WVTR of PSS and plasticized PSS films. Glass bottles containing silica gel were covered with the films and the bottles were placed in a desiccator which was filled with a saturated NaCl solution (75% relative humidity). The bottles were weighed every hour to record the weight change until the sample weight was stable. The WVTR was calculated using Equation (3).

(3) $$\mathrm{WVTR}=\frac{w_{f}-{\;w}_{i}}{\mathrm{day}\;\times A}\times 1$$

where w_i_ is the initial bottle weight and w_f_ is the bottle weight after the test each day. The weight difference each day was plotted against time (day). *A* is the effective film area in m^2^ and l is the film thickness for normalization.

Wettability: the water contact angles of the films were measured to determine the wettability of the film surface. Deionized (DI) water was dropped on the film surface manually and the droplet shape was recorded with a high magnification microscope. Then the contact angle was determined using an Image J program.

Banana coating: Green bananas were obtained from a local market. Starch solutions containing different glycerol contents were prepared following the method described in Section 2.2.2. The solutions were then sprayed onto bananas with a commercial spray gun. The coated bananas were left in laboratory ambient conditions and monitored daily.

## 3. Result and Discussion

### 3.1. Film Appearance and Transparency

**Figure 1:**
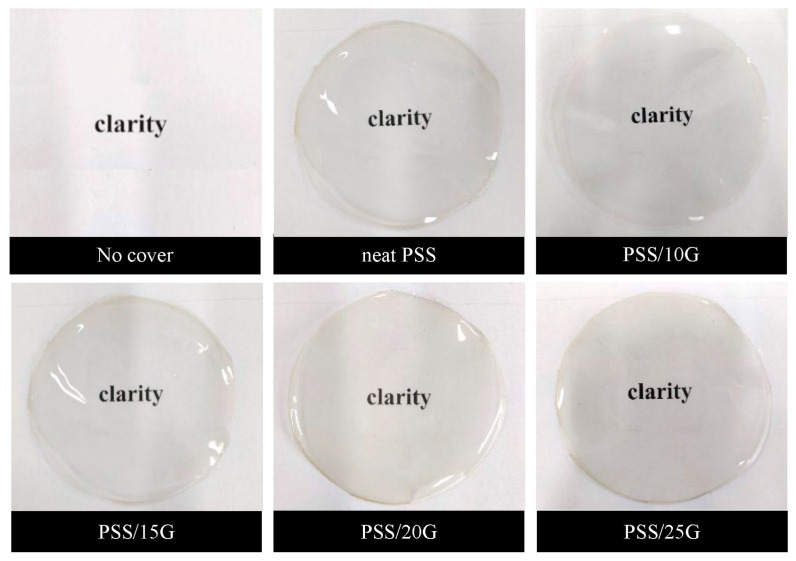
Appearance of neat PSS and plasticized PSS films with different glycerol contents.

**Figure 2:**
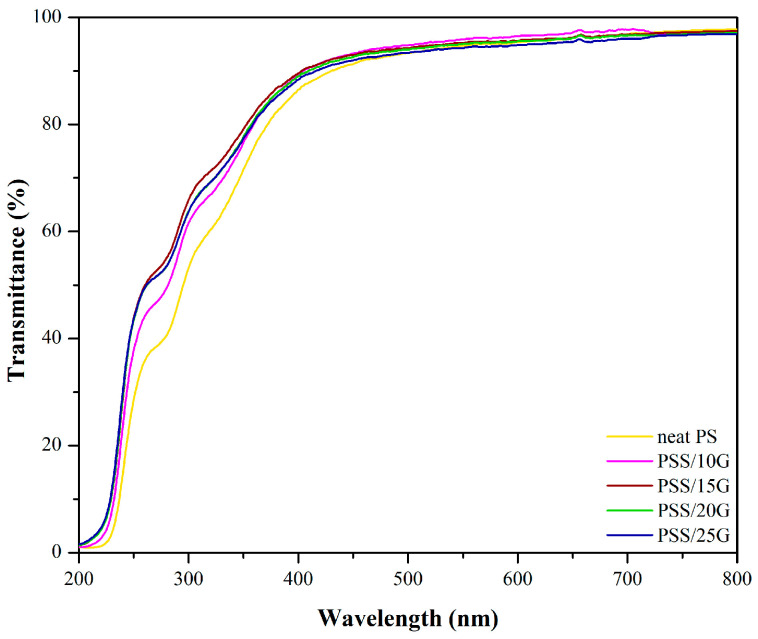
Transmittance spectra of neat PSS and plasticized PSS films with different glycerol contents.

Photographs of the PSS and plasticized PSS films are shown in Figure 1. All films display a very good appearance after removing form their molds illustrating that all the films peel off well. Moreover, increase of glycerol content allows the films to be peeled more readily. In addition, these films are very clear and shiny. Figure 2 displays the transparency of these films with a high percentage of transmittance of around 95% at wavelength 550 nm in the visible region. Furthermore, the addition of glycerol has little effect on the transparency of the PSS films. Thus, in terms of film transparency, these PSS films are acceptable as a food coating material.

### 3.2. X-ray Diffraction

**Figure 3:**
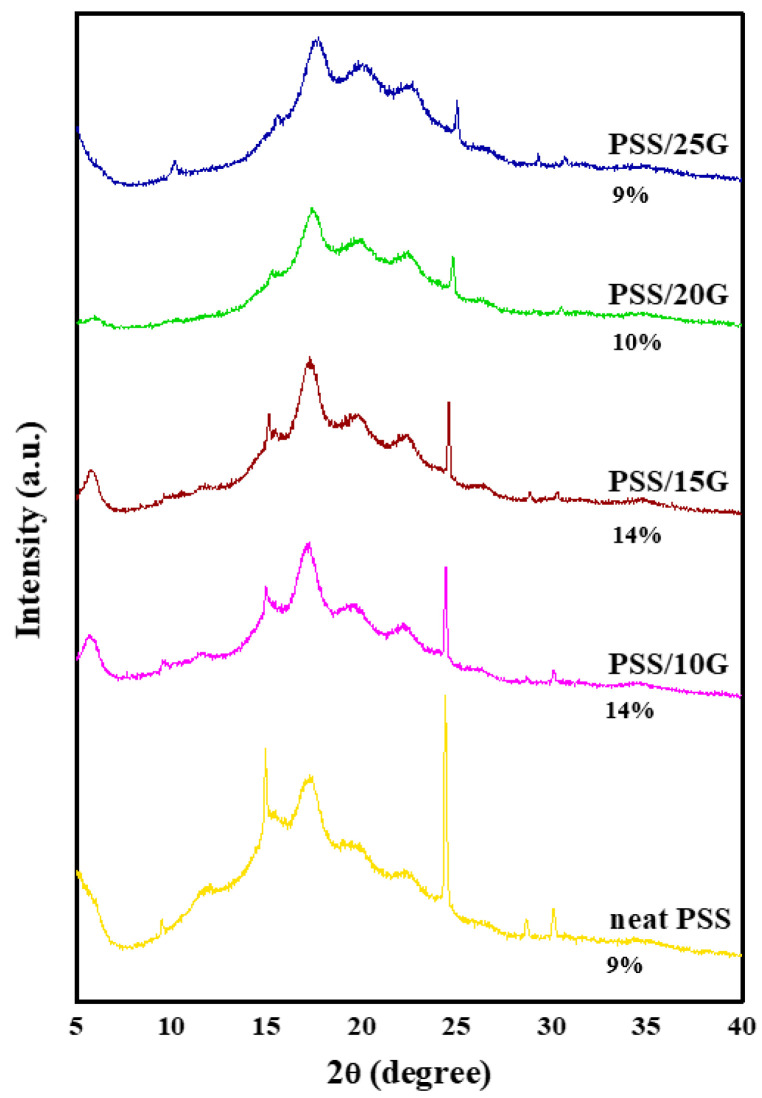
X-ray diffraction patterns of neat PSS and plasticized PSS films with different glycerol contents. The numbers in the graph indicate the crystallinity of the films.

The X-ray diffraction patterns of the PSS and plasticized PSS films are shown in Figure 3. All films display characteristic broad peaks at 17.6°, 19.9° and 22.3°. There are sharp peaks at 14.9° and 24.3° which could be some contaminants. The results indicate that PSS starch is a semi-crystalline polymer which exhibits B-type crystal pattern following its retrogradation [39,40]. Glycerol content has no influence on the crystal type of PSS films as the same diffraction pattern is observed. However, a slight reduction of peak intensity at 14.9° and 24.3° has taken place with increasing glycerol content (PSS/20G and PSS/25G). This is presumably due to the plasticizing effect of glycerol between the starch chains causing the reduction in crystallinity of the films. This demonstrates that glycerol was able to mix in the starch matrix, so the crystallization was hindered, and the crystallinity was decreased.

### 3.3. Mechanical Properties

**Figure 4:**
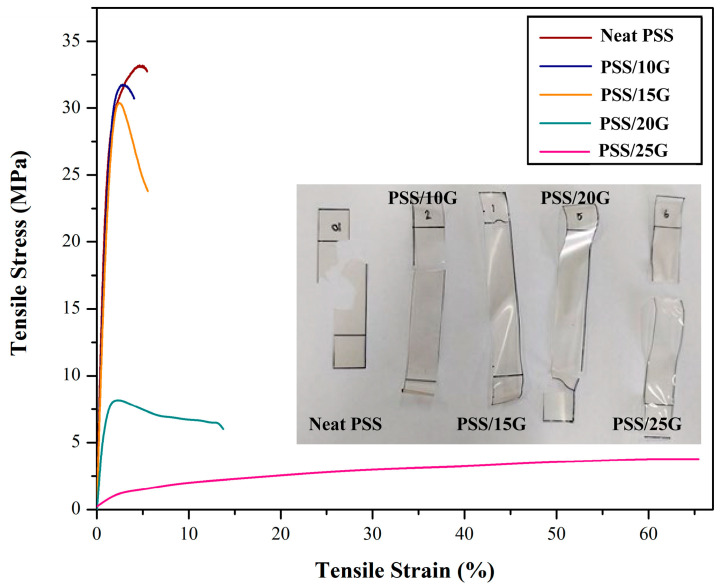
Mechanical properties of neat PSS and plasticized PSS films with different glycerol contents. Insert is photographs of different films after tensile test.

**Figure 5:**
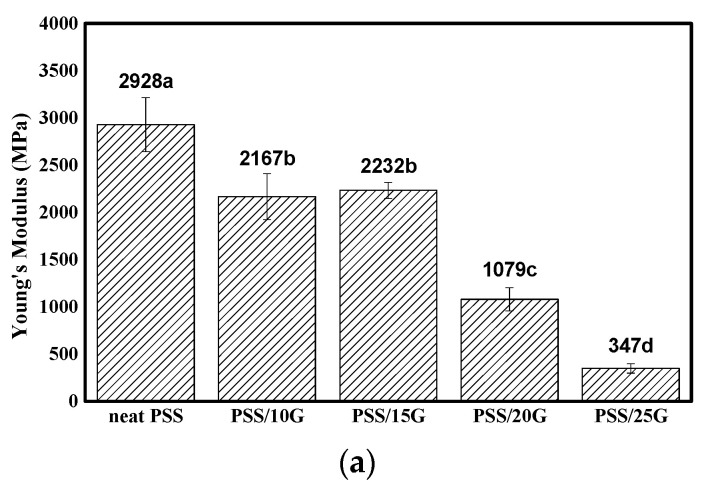

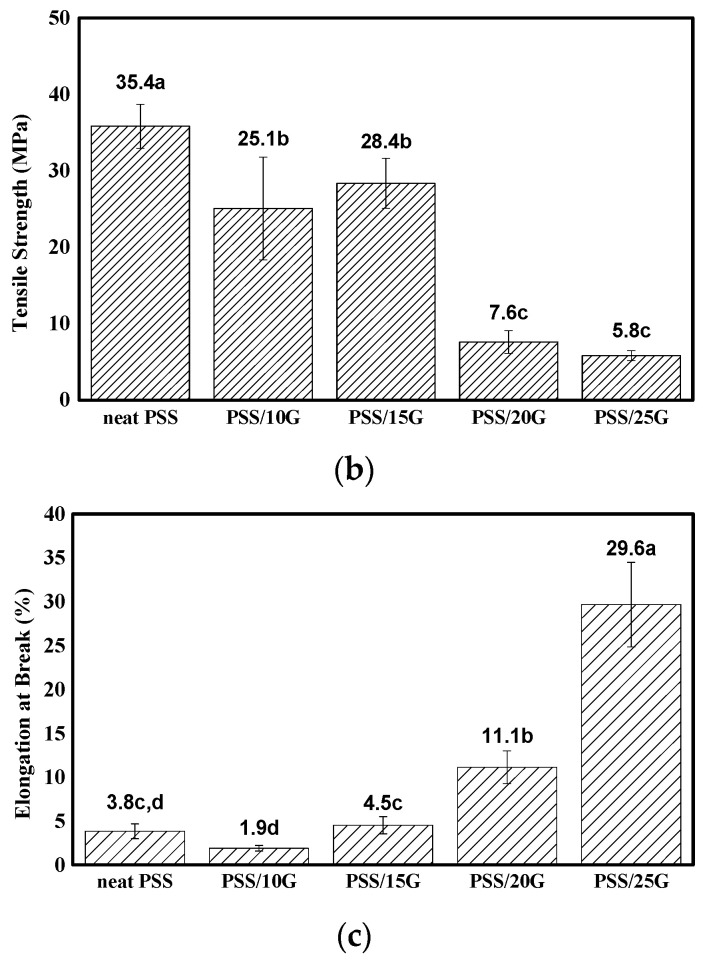
Mechanical properties of neat PSS and plasticized PSS films with different glycerol contents: (**a**) Young modulus, (**b**) tensile strength and (**c**) elongation at break. Different letters on each bar indicate significant differences (determined using a paired *t*-test, *p* < 0.0001).

Representative stress-strain curves of the PSS and plasticized PSS films are shown in Figure 4. PSS and plasticized PSS films with glycerol contents equal to or less than 10 wt.% exhibit high tensile strength and low extensibility. Although it was not visible on the samples, the stress-strain curves showed evidence of yielding. Only when the glycerol content was increased to equal to or more than 20 wt.% was yielding observed on the tested samples. Average values for moduli, tensile strengths and elongation at break are shown in Figure 5. The tensile strength of PSS film is presumably due to its high amylose content [41]. It is slightly lower than that of mung bean starch [42] but higher than that of high amylose rice starch [43] and cassava starch [44]. This is possibly related to the fact that mung bean starch has a higher amylose content of about 40.7% [42] while high amylose rice starch and cassava starch have amylose contents of about 30.4% and 15.4%, respectively. The modulus and tensile strength of the films decrease with increasing glycerol content while elongation at break increases. It is interesting to note that the amount of glycerol at which the tensile strength starts to drop and elongation at break starts to increase is about 20 wt.% which is slightly lower than that observed in mung bean systems [42].

### 3.4. Morphology

**Figure 6:**
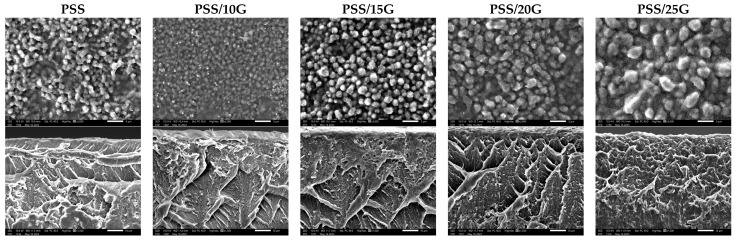
SEM images of neat PSS and plasticized PSS films with different glycerol contents; air exposed surface (top row) and fractured surface (bottom row).

Figure 6 displays the air-exposed surfaces and internal structure of PSS and plasticized PSS films with varying glycerol contents. The air-exposed film surfaces exhibit a granular structure, similar to what has been previously reported by our group [36] for PSS films with glycerol and citric acid, as well as by Liu [45] for high-amylose films. The size of these granules ranges from 1 to 5 μm, and their size appears to increase with higher glycerol content. These granules are smaller compared to the initial PSS granule size of approximately 10 μm [35]. Hence, it can be inferred that this granular structure forms during the retrogradation process, with the increased glycerol content allowing starch molecules to become more mobile, leading to the formation of larger structures.

Another notable observation pertains to the internal structure of these films. In contrast to the thicker films [36] and sheets [46] recently reported, our films do not exhibit a granular or porous structure as observed in those respective specimens. This difference is likely due to the relatively lower concentration of starch solution used for film casting, resulting in thinner films that dry quickly. Thicker films would require longer drying times, allowing for retrogradation and the development of a granular structure. Moreover, for sheets, the slower water evaporation would facilitate the syneresis process, whereby water is expelled from the starch network due to retrogradation, resulting in void formation upon water evaporation [47].

Further investigation into the nature of this granular structure is currently under way and will be reported in future correspondence.

### 3.5. Gas Transmission Rate

**Figure 7:**
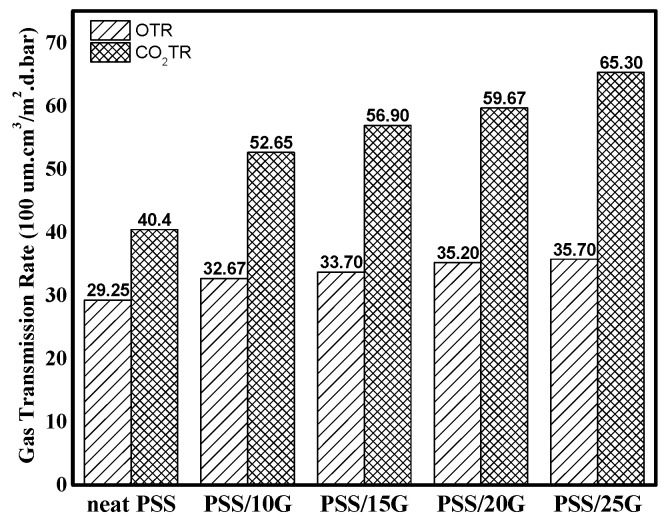
Gas transmission rates (oxygen and carbon dioxide) of neat PSS and plasticized PSS films with different glycerol contents.

Figure 7 shows the oxygen and carbon dioxide transmission rates of the PSS and plasticized PSS films. As the glycerol content was increased, both the carbon dioxide and oxygen transmission rates also increased, with carbon dioxide permeating the films more quickly than oxygen. Glycerol content has a greater influence on the transmission rate of carbon dioxide than that of oxygen. This result agrees well with the general observation that carbon dioxide diffuses between two to five times faster than oxygen [48]. The transmission rate of carbon dioxide increases at a rate that is roughly 2.7 times that of oxygen with the addition of around 25 wt.% of glycerol. This is due to the higher polarity of the bonds in carbon dioxide molecules, allowing greater interaction with polar hydroxyl groups on the starch molecules.

### 3.6. Water Vapor Transmission Rate

**Figure 8:**
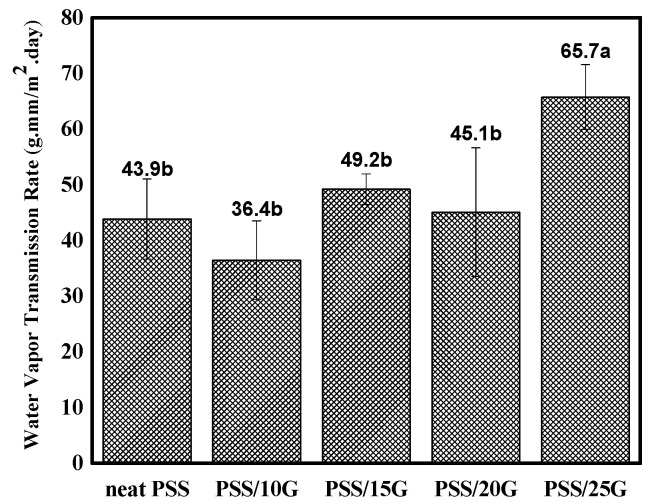
Water vapor transmission rate of neat PSS and plasticized PSS films with different glycerol contents. Different letters on each bar indicate significant differences (determined using a paired *t*-test, *p* < 0.0001).

The water vapor transmission rates of the PSS and plasticized PSS films are shown in Figure 8. The WVTR is around 45 g.mm/m^2^.day for PSS and plasticized PSS with glycerol contents equal to and below 20 wt.%. The WVTR started to increase by about 45% when glycerol content was increased to 25 wt.%. These values are much less than those of other starch films (cf. [49,50,51]) and this should be related to the crystalline nature of PSS. When the glycerol content increased, the crystallinity decreased as shown in Section 3.2, leading to higher WVTR.

### 3.7. Wettability

**Figure 9:**
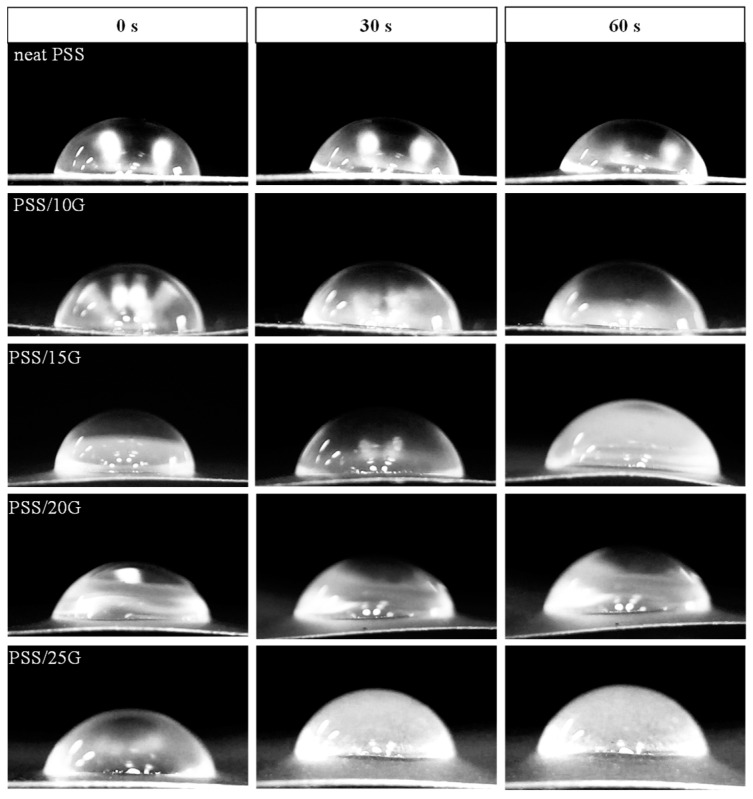
Water contact angle on the surface of neat PSS and plasticized PSS films with different glycerol contents.

The water wettability and water resistance of the films were evaluated by measuring the water contact angle on the film surface and observing the changes in droplet shape over time. The water droplet shapes can be seen in Figure 9, and the corresponding numerical data for contact angles are presented in Table 2. The neat PSS film exhibited a water contact angle of approximately 80°. With an increase in the amount of glycerol, there was a slight decrease in the contact angle. In addition, the contact angle showed a slight decrease as the water droplet remained on the film surface for a longer duration. Importantly, all the films maintained their shape and integrity over time, demonstrating their ability to resist water absorption, unlike many starch films. These findings suggest that the films exhibit a certain degree of water resistance and can effectively resist water penetration.

### 3.8. Preliminary Test for Banana Coating

**Figure 10:**
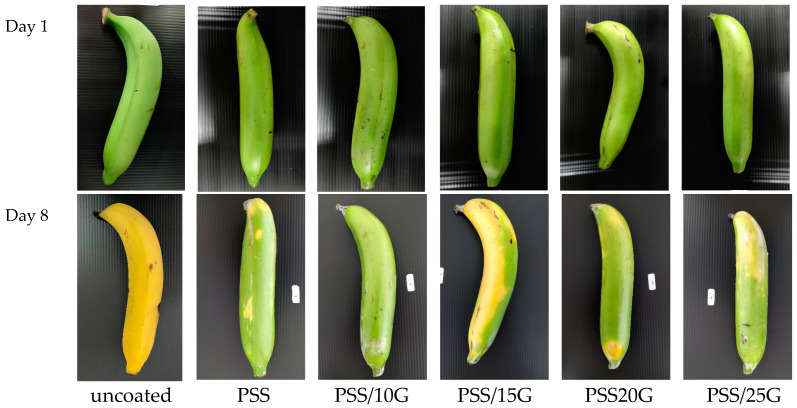
Photographs of bananas that were coated with PSS and plasticized PSS films before and after being left at ambient condition for the indicated period of time.

The banana is one type of fruit that is widely studied due to its short shelf life [52], and different types of coating have been studied [17]. It has been shown that if gas transmission rates (water vapor, oxygen and carbon dioxide) can be modulated with a coating, shelf life can be extended [17,52]. Thus, the fact that the PSS films display good water resistance and adjustable transmission rates for different gases has prompted us to test the film as a coating for prolonging the ripening process of bananas. To demonstrate this potential, simple starch solutions containing different amount of glycerol were prepared and the solution was sprayed onto bananas. The results are shown in Figure 10. The obtained results have shown that, after one week, the control banana had turned completely yellow while all the coated bananas were still green and only some places just started to turn yellow. Bananas that were coated with films with higher glycerol contents displayed more yellow patches. The non-uniform ripening could be due to the uneven coating thickness on the fruit. Work is now under way to improve the formulation of the solution and the method for achieving a uniform and effective coating.

Despite limited results, the aforementioned demonstration showed that pineapple stem starch has a great potential for use in coating fruits and vegetables. By altering the glycerol concentration or film thickness, gas transfer rates for each variety of product can be tailored to match its transpiration and respiration properties. Moreover, to create an active coating for even greater performance, active agents such as ethylene absorbers, moisture absorbers, oxygen scavengers, etc. could be included in the starch matrix, for example [5,6,7,9,17]. This will be the topic of future research in order to make good use of pineapple stem starch as a coating material.

## 4. Conclusions

PSS was used to create flexible, water-resistant films with a variety of mechanical characteristics and gas transmission rates (oxygen, carbon dioxide and water vapor). Due to the high amylose content of the starch that gives the films their water resistance, the films display some crystallinity. By adjusting the amount of glycerol, the mechanical characteristics and gas transfer rates could be controlled. As the glycerol content was increased, the films became softer, more extensible and more gas permeable. The bananas coated with the film-forming PSS solution demonstrated that ripening can be delayed for longer than a week. These materials hold enormous promise as a coating for fruits and vegetables. This would not only help turn leftover pineapple stems into a valuable commodity but also reduce food waste and improve food security.

## Figures and Tables

**Table 1:** Codes and composition of neat PSS and plasticized PSS films.

Sample	Starch (wt.%)	Glycerol (wt.%)
neat PSS	100	0
PSS/10G	90	10
PSS/15G	85	15
PSS/20G	80	20
PSS/25G	75	25

**Table 2:** Water contact angle of neat PSS and plasticized PSS films with different glycerol contents.

Sample	Water Contact Angle (Degree)		
	0 s	30 s	60 s
neat PSS	80.1 ± 0.5 ^a^	76.0 ± 0.6 ^a^	72.0 ± 1.5 ^b^
PSS/10G	79.7 ± 0.6 ^a^	76.0 ± 0.4 ^a^	73.8 ± 0.8 ^a^
PSS/15G	79.9 ± 0.7 ^a^	76.9 ± 1.0 ^a^	74.1 ± 0.3 ^a^
PSS/20G	75.4 ± 1.2 ^b^	74.2 ± 1.3 ^b^	71.4 ± 0.5 ^b^
PSS/25G	72.5 ± 0.7 ^c^	70.5 ± 0.3 ^c^	70.7 ± 0.3 ^b^

Different superscript letters for each column indicate significant differences (determined using a paired *t*-test, *p* < 0.0001).

## Data Availability

Data is contained within the article.
